# Adaptation to Chronic Nutritional Stress Leads to Reduced Dependence on Microbiota in *Drosophila melanogaster*

**DOI:** 10.1128/mBio.01496-17

**Published:** 2017-10-24

**Authors:** Berra Erkosar, Sylvain Kolly, Jan R. van der Meer, Tadeusz J. Kawecki

**Affiliations:** a Department of Ecology and Evolution, University of Lausanne, Lausanne, Switzerland; b Department of Fundamental Microbiology, University of Lausanne, Lausanne, Switzerland; EPFL

**Keywords:** adaptation, digestion, *Drosophila*, experimental evolution, juvenile development, microbiota, nutritional stress, dFOXO

## Abstract

Numerous studies have shown that animal nutrition is tightly linked to gut microbiota, especially under nutritional stress. In *Drosophila melanogaster*, microbiota are known to promote juvenile growth, development, and survival on poor diets, mainly through enhanced digestion leading to changes in hormonal signaling. Here, we show that this reliance on microbiota is greatly reduced in replicated *Drosophila* populations that became genetically adapted to a poor larval diet in the course of over 170 generations of experimental evolution. Protein and polysaccharide digestion in these poor-diet-adapted populations became much less dependent on colonization with microbiota. This was accompanied by changes in expression levels of dFOXO transcription factor, a key regulator of cell growth and survival, and many of its targets. These evolutionary changes in the expression of dFOXO targets to a large degree mimic the response of the same genes to microbiota, suggesting that the evolutionary adaptation to poor diet acted on mechanisms that normally mediate the response to microbiota. Our study suggests that some metazoans have retained the evolutionary potential to adapt their physiology such that association with microbiota may become optional rather than essential.

## IMPORTANCE

Animals depend on gut microbiota for various metabolic tasks, particularly under conditions of nutritional stress, a relationship usually regarded as an inherent aspect of animal physiology. Here, we use experimental evolution in fly populations to show that the degree of host dependence on microbiota can substantially and rapidly change as the host population evolves in response to poor diet. Our results suggest that, although microbiota may initially greatly facilitate coping with suboptimal diets, chronic nutritional stress experienced over multiple generations leads to evolutionary adaptation in physiology and gut digestive properties that reduces dependence on the microbiota for growth and survival. Thus, despite its ancient evolutionary history, the reliance of animal hosts on their microbial partners can be surprisingly flexible and may be relaxed by short-term evolution.

## INTRODUCTION

Nutrient availability is a major factor limiting survival, growth, and reproduction of many animal species (1), resulting in natural selection for adaptation to cope with nutritional stress. Yet, little is known about evolutionary adaptations that help juvenile animals not only to survive but also to grow, develop, and reach maturity under chronic nutrient shortage. Recent studies point to a particular importance of gut microbiota in coping with such chronic nutritional stress. For example, monocolonization with *Lactobacillus plantarum* protects the growth of infant mice against the effects of nutrient shortage through a mechanism involving insulin-like growth factor (IGF) signaling (2). Important insights about the mechanisms of microbiota-mediated enhancement of fitness under nutrient shortage have recently emerged from studies in *Drosophila melanogaster*. Like other insects that feed on a variety of sources, *Drosophila* has a rather simple and transient gut microbiota consisting of a subsample of ambient bacteria growing on its food (decomposing fruits) (3). Nonetheless, similarly to the more specialized commensals of mammals, these microbes provide a number of nutritional and metabolic benefits to their hosts (4, 5). The same strain of *L. plantarum* that alleviates the effect of nutrient limitation on growth of mice (2) promotes the growth of *Drosophila* larvae on a protein-poor diet. This effect is mediated through upregulation of the host’s proteolytic enzymes, leading to enhanced digestion and modulation of insulin and TOR pathways (6, 7). Another commensal, *Acetobacter pomorum*, was also found to promote *Drosophila* larval growth by modulating insulin/IGF-like signaling (IIS); this phenotype was again particularly pronounced on poor diets (8). Based on these findings, one might hypothesize that animal populations often exposed to chronic malnutrition would adapt by evolving an improved ability to benefit from their microbiota.

We address this hypothesis with experimental evolution (i.e., the study of evolutionary processes occurring in experimental populations in response to conditions imposed by the experimenter) (9). To study genetically based evolutionary adaptation to chronic juvenile nutritional stress, we have maintained six outbred *Drosophila melanogaster* populations (“Selected” populations) for over 170 generations on an extremely poor larval diet (containing only 0.3% [wt/vol] yeast). The nutrient content of the poor diet is so low that nonadapted larvae take twice as long to develop as on a standard diet (which contains 1.2% [wt/vol] yeast), and the resulting adults are only half of normal weight (10). As there was no gene flow between these populations, they represent independent replicate instances of evolutionary change driven by the poor food regime. Compared to six “Control” populations maintained in parallel on a standard diet, the Selected populations evolved increased egg-to-adult survival, smaller critical size for metamorphosis initiation, and faster development on the poor diet (10, 11).

Here, we test how this enhanced performance of the poor-diet-adapted Selected populations depends on interactions with microbiota and study the underlying physiological mechanisms. By manipulating the microbiota colonization status of larvae, we demonstrate that, contrary to our expectations, these poor-diet-adapted populations became less dependent on microbiota for growth and survival on the poor diet. We show that protein and carbohydrate digestion in Selected larvae is much less affected by microbiota than in Controls, in spite of the two types of larvae carrying microbiota of similar compositions and abundances. Finally, our populations exhibit differential expression of major cell growth regulator dFOXO (12) and many of its targets. This indicates that the site-specific function of dFOXO contributes to the physiological changes underpinning adaptation to nutritional stress, paralleling the microbiota effect in the nonadapted Control populations.

## RESULTS

### Effect of microbiota on development and survival of experimentally evolved populations.

**FIG 1:**
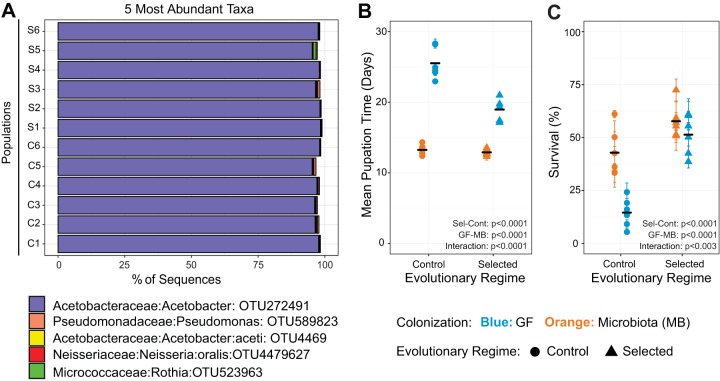
Microbiota affects development and survival differently in Selected and Control populations on poor diet. (A) Identities and relative abundances of the 5 most abundant taxa in Selected and Control larvae reared in their conventional media, assigned by 16S rRNA gene amplicon sequencing. (B) Mean egg-to-pupa development time in Selected and Control populations, with or without microbiota. (C) Mean egg-to-pupa survival rate under the same conditions. Symbols and error bars represent means ± standard errors of the means for each population (where error bars are not visible, they are smaller than the symbols). Black horizontal bars represent the means for the six replicate populations. Main effect differences analyzed by GMM are represented in the panels. Interaction = colonization × regime. Detailed statistics are presented in Table S1 in the supplemental material.

To assess the effect of microbiota on growth of animal hosts genetically adapted to nutritional stress, we used experimentally evolving Selected and Control populations that were bred on a poor and a standard diet, respectively, for over 170 generations (i.e., over the course of 10 years [see Materials and Methods]). To control the larval density, fly eggs were rinsed and counted in a nonsterile environment at each generation (see Materials and Methods for details), which hindered vertical transmission of microbiota within populations from one generation to the next and was conducive to exchange of microbes between populations as well as with the general environment of the incubator. Therefore, we did not expect one-to-one coevolution between the populations and their specific microbial communities. To validate this assumption, we performed 16S rRNA gene sequencing on larvae that were collected from their respective breeding diets (i.e., larvae of Selected populations from poor diet and larvae of Control populations from standard diet). We found that a single *Acetobacter* sp. strongly dominated (>95% abundance) the gut communities of all Control and Selected populations irrespective of diet and evolutionary history of the populations (Fig. 1A). This confirmed that the Selected and Control populations did not develop different microbiota communities Therefore, any differences in responses between Selected and Control populations to microbiota would be unlikely to be due to differences in the microbiota communities with which they coevolved; instead, they would be driven by adaptation to different dietary regimes in the presence of similar microbiotas.

10.1128/mBio.01496-17.3 TABLE S1  Detailed statistics for data presented in Fig. 1B and C. Download TABLE S1, PDF file, 0.02 MB. Copyright © 2017 Erkosar et al. 2017 Erkosar et al. This content is distributed under the terms of the Creative Commons Attribution 4.0 International license.

As a consequence of evolutionary adaptation to poor diet over 170 generations, larvae of our Selected populations had previously been reported to develop faster and survive better than Control larvae on a poor diet (but not on a standard diet) (10, 13). However, in those studies the colonization of the larvae by microbiota was not controlled and not assessed. As explained in the introduction, our study was motivated by the hypothesis that the improved performance of Selected larvae on the poor diet is at least in part mediated by an improved ability of larvae to benefit from interactions with microbiota. If so, one would predict that their superiority over Control larvae would diminish if they were deprived of the help of microbiota, i.e., in a germ-free (GF) state. To test this prediction, we compared the lengths of larval development and survival rates of Selected and Control populations when experimentally colonized with microbiota (MB) with those in a GF state. The experimental colonization was performed by fecal transplantation with a common microbiota inoculum collected from the feces of adults of all 12 populations (added on sterile eggs to colonize freshly hatching larvae). The GF state was obtained by supplementing sterile eggs with a heat-killed inoculum (to control for potential effect of bacteria as food [see Materials and Methods for details]).

Contrary to our prediction, the microbiota affected the Control populations much more strongly than the Selected populations. Whereas Control larvae colonized with microbiota developed 40% faster and were three times more likely to survive on the poor diet than their GF siblings, the corresponding effect of microbiota treatment on Selected larvae was much smaller (Fig. 1B and C; for statistical analysis see Table S1 in the supplemental material). As a consequence, the difference between Selected and Control populations was much less pronounced in the colonized than in the GF state (Fig. 1B and C). In particular, the developmental time became statistically indistinguishable between the Selected and Control larvae in the colonized state (even though Selected larvae still tended to develop about 1 day faster than Controls). These results are contrary to our hypothesis and imply that, in the course of their evolutionary adaptation to poor diet, the Selected populations became less dependent on microbiota and much better able to cope with nutrient shortage without their help.

### Protein digestion in Selected and Control populations.

It has been shown previously that on nutritionally poor diets one of the members of the *Drosophila* microbiota, *Lactobacillus plantarum*, promotes intestinal protease expression, leading to enhanced dietary protein digestion and increased amino acid concentrations in host tissues (7). We therefore hypothesized that the weaker effect of microbiota on the survival and developmental time of Selected than of Control populations could be mediated by differential effects on protein digestion.

**FIG 2:**
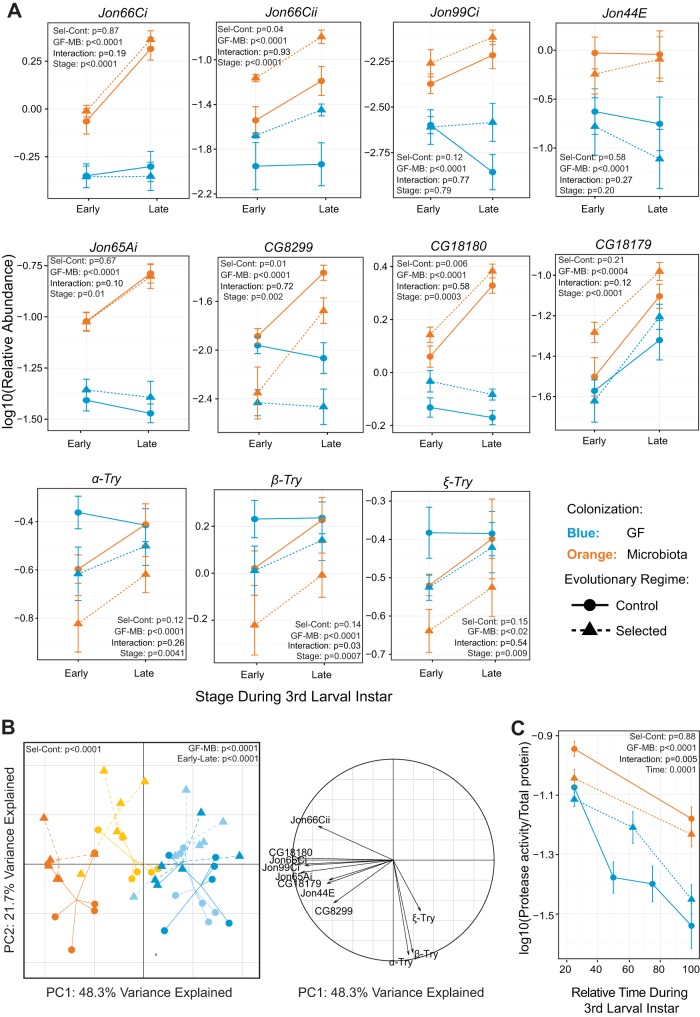
Microbiota affects protein digestion differently in Selected and Control populations. (A) Relative expression (2^−Δ*CT*^) of different proteases measured by qRT-PCR from dissected guts of Selected and Control larvae at early and late L3 stage. Symbols represent means ± standard errors of the means for the six replicate populations, with 3 biological replicates per population. A selection of key statistical results from GMM is represented in the panels. Interaction = colonization × regime. Detailed statistics, including pairwise contrasts, are presented in Table S2. (B) Projections of protease expression data set into first and second PCs (left) together with correlation circle (right) representing the variables. Light shading, early third instar; dark shading, late third instar. (C) Protease activity in Selected and Control larvae in the course of the third larval instar in the presence or absence of microbiota.

Seven serine proteases, including five proteases of the Jonah family, have been reported to be transcriptionally induced upon colonization with *L. plantarum* (7). We decided to verify if expression of the same proteases is induced by the microbiota of our larvae (even though it was dominated by *Acetobacter* rather than *Lactobacillus*) and if this induction differs between the Selected and Control populations, potentially contributing to the differences in their development and survival on the poor diet. To this end, we dissected the intestines of GF and microbiota-colonized larvae at early and late third instar and used reverse transcription-quantitative PCR (qRT-PCR) to quantify the expression of 11 proteases, including trypsins, Jonah proteases, and a few others known to have serine-type protease activity. Consistent with the previous report (7), we detected a higher expression in colonized than in GF larvae of all five Jonah proteases and three other serine proteases (*CG18179*, *CG18180*, and *CG8299* [orange versus blue symbols in Fig. 2A]). In contrast, trypsin superfamily proteases (*α-Try*, *β-Try*, and ε-*Try*, which are clustered together in the genome and reported to have a very localized expression in the gut [14]) were downregulated by microbiota compared to the GF state (Fig. 2A, orange versus blue symbols). Out of the 11 proteases, we identified two (*Jon66Cii* and *CG18180*) whose mRNA levels were consistently significantly higher in Selected populations than in Controls; we also observed that *CG8299* had higher expression in Control than in Selected populations (Fig. 2A). Trends for differences between Selected and Control populations could also be observed for several other proteases (all three trypsins, *CG18179*, *Jon65Ai*, *Jon44E*, and *Jon99Ci* [Fig. 2A]), but they were not sufficiently consistent between time points or replicate populations to be statistically significant. The digestive proteases are likely to some degree functionally redundant, and thus, it is conceivable that evolution would achieve functionally similar changes in digestion by targeting different genes in different replicate populations, making detection of a signature of evolution in a gene-by-gene analysis difficult. Therefore, we used two complementary approaches to address differences in protein digestion globally rather than on a gene-by-gene basis.

10.1128/mBio.01496-17.4 TABLE S2  Detailed statistics for data presented in Fig. 2A and C. Download TABLE S2, PDF file, 0.1 MB. Copyright © 2017 Erkosar et al. 2017 Erkosar et al. This content is distributed under the terms of the Creative Commons Attribution 4.0 International license.

First, we analyzed the qRT-PCR protease expression data set with multivariate analysis of variance (MANOVA) and principal-component analysis (PCA). The correlation circle clearly confirmed that the levels of expression of the three trypsins were positively correlated and well separated from other proteases (Fig. 2B, right). This suggests that these two groups of proteases are regulated by different processes and/or may have different functions within the gut. GF and microbiota-colonized larvae were clearly separated by the first PC, with Selected and Control populations somewhat less distinctly separated along the second PC (Fig. 2B, left). Given that the first PC explains more than twice as much variance as the second does, this implies that microbiota are a major factor that changes protease expression, with a greater impact than the evolutionary history of adaptation to poor versus standard diet. Nonetheless, the impact of both microbiota colonization and evolutionary adaptation was highly significant in the MANOVA (*P* < 0.0001; for details see Table S2). Interestingly, the MANOVA detected no interaction between the effects of microbiota colonization and those of the evolutionary regime (*P* = 0.30, Table S2). This means that although Selected and Control populations differed in the patterns of protease expression, the effects of microbiota on protease expression were similar in the two sets of populations.

Second, as a readout of combined effects of the abundance and catalytic activity of different proteases, we measured protease activity (relative to the total protein content) in whole GF and microbiota-colonized larvae at different time points during the third instar (Fig. 2C; see Materials and Methods for details). This relative protease activity declined over time, which could reflect changes in protease secretion as well as an increase in the amount of protein accumulated in the larval body relative to the size of the digestive system. Irrespective of this apparent decline over time, colonization by microbiota strongly enhanced proteolysis in Control larvae but had a significantly smaller effect on proteolysis in Selected larvae (as evidenced by the significant interaction term in Fig. 2C, *P* = 0.005). While GF Selected larvae exhibited (marginally significantly, *P* = 0.083) higher levels of protease activity than GF Controls, the trend went in the opposite direction in microbiota-associated larvae. This pattern of proteolytic activity of the Selected and Control larvae in the absence and presence of microbiota matches qualitatively the pattern of larval developmental rate and survival reported above (Fig. 1B and C). Together with the known role of enhanced proteolysis in mediating microbiota effects on larval growth on poor diet (7), these results support the notion that the improvement in larval performance, whether owing to microbiota or to evolutionary adaptation, is at least in part mediated by enhanced protein digestion efficiency.

### Carbohydrate digestion in Selected and Control populations.

**FIG 3:**
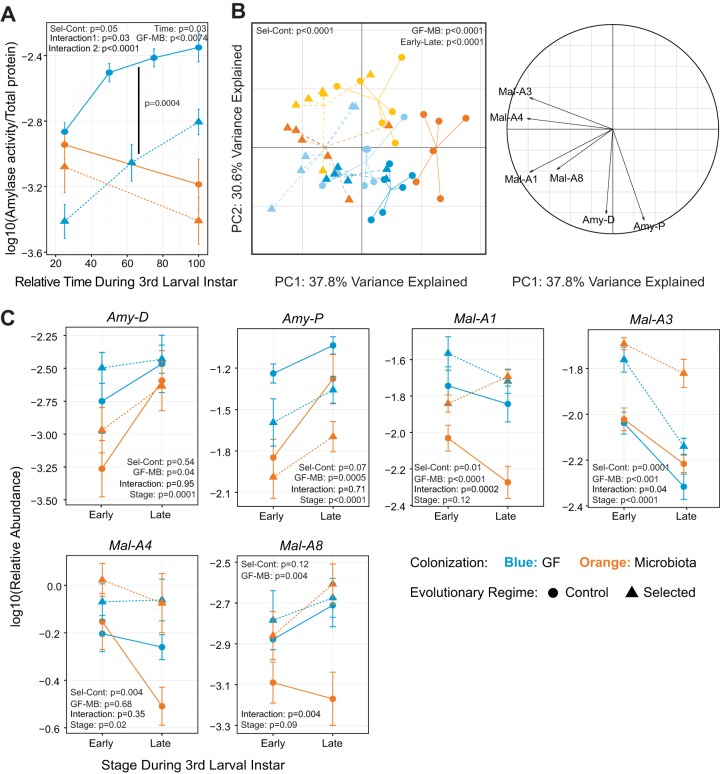
Microbiota affects carbohydrate digestion differently in Selected and Control populations. (A) Amylase activity in Selected and Control larvae throughout the third larval instar in the presence or absence of microbiota (MB). Significant pairwise differences between GF Control and GF Selected populations are shown with a black line. (B) Projections of amylase and maltase expression data set into first and second PCs (left) together with correlation circle (right) representing the variables. Light shading, early third instar; dark shading, late third instar. (C) Relative expression (2^−Δ*CT*^) of different amylases and maltases measured by qRT-PCR from dissected guts of Selected and Control larvae at early and late third instar. Symbols represent means ± standard errors of the means for the six replicate populations, with three biological replicates each. A selection of key statistical results from GMM is represented in the panels. Interaction = colonization × regime; interaction 2 = time × colonization. Detailed statistics, including pairwise contrasts, are presented in Table S3.

Given that our poor diet is low in carbohydrate as well as protein content, we next asked if carbohydrate digestion is also different between Selected and Control populations and if it is differentially influenced by microbiota. About 30% of carbohydrates in both poor and standard diets consist of polysaccharides (starch) from the cornmeal (the rest are sucrose and glucose). Polysaccharide digestion occurs as a two-step process whereby starches are first broken down to disaccharides by amylases before being hydrolyzed to monosaccharides. Alpha-amylase activity is under direct negative regulation by glucose concentration in *Drosophila* larvae, which occurs at the transcriptional level. Amylase activity is therefore expected to be lower in larvae with higher glucose concentrations (15, 16). We quantified amylase activity rates (again normalized to total larval protein content) in Selected and Control larvae in both colonized and GF states. Microbiota had a striking effect on how amylase activity changed over time: while it declined between early and late third stage in the microbiota-colonized larvae, it increased sharply during the corresponding developmental period in GF larvae (slope difference, *P* < 0001) (Fig. 3A). Because no such increase was observed for protease activity (Fig. 2C), it implies that GF larvae upregulate their investment in polysaccharide digestion relative to protein digestion toward the end of their development. Irrespective of these temporal changes, GF Selected larvae consistently showed 3-fold-lower amylase activity than GF Control larvae of the same stage (blue symbols in Fig. 3A); this difference is much smaller and nonsignificant in microbiota-colonized larvae (orange symbols in Fig. 3A). Thus, we again observed a pattern of interaction such that the difference due to evolutionary history was more pronounced in the germ-free than in the microbiota-colonized state. Interestingly, fast development and high survival on a poor diet (Fig. 1B and C) were associated with lower amylase activity. This implies that increased amylase activity is a sign of nutritional stress. Given the negative regulation of amylase activity by glucose concentration (15, 16), these results suggest that Control larvae maintain lower glucose levels than Selected larvae under GF conditions.

10.1128/mBio.01496-17.5 TABLE S3  Detailed statistics for data presented in Fig. 3A and C. Download TABLE S3, PDF file, 0.1 MB. Copyright © 2017 Erkosar et al. 2017 Erkosar et al. This content is distributed under the terms of the Creative Commons Attribution 4.0 International license.

To verify if the pattern that we observed is regulated at the transcriptional level, we quantified amylase transcript levels in the guts. We analyzed expression of two amylases. Both gene transcripts were significantly reduced in microbiota-colonized larvae in all populations (Fig. 3C). Under GF condition, *Amy-P* levels were higher in Control populations than in Selected populations, but no significant difference was detected in *Amy-D* levels (Fig. 3C). Given that the transcript abundance of *Amy-P* was roughly 20-fold higher than that of *Amy-D* (Fig. 3C), *Amy-P* is likely to be the major gene contributing to the amylase activity pattern that we observed earlier (Fig. 3A). Even though *Amy-P* expression is reduced by microbiota and *Amy-P* is expressed at lower levels in Selected than in Control populations, the expression pattern does not fully explain the pattern of amylase activity, suggesting involvement of other regulatory mechanisms (e.g., cAMP levels [15]).

In the gut, glucose is generated through the hydrolysis of maltoses by maltases. If amylase activity is lower in Selected than Control populations and upon microbiota colonization than in the GF state because of glucose concentration in the gut and/or hemolymph, maltase activity is predicted to be higher under these conditions. To check this, we also analyzed expression of four maltase genes. In agreement with this prediction, we observed a high expression of maltases in Selected populations for *Mal-A1*, *Mal-A3*, and *Mal-A4*, although not for *Mal-A8* (Fig. 3C). A consistent decrease in expression can be observed upon colonization only in Control populations for *Mal*-*A1* and *Mal-A8* (Fig. 3C). *Mal-A4* exhibits this trend only at late third instar, but this is not statistically significant, due to high variation among populations (Fig. 3C). *Mal-A3* expression is rather induced in Selected populations upon colonization and remains unchanged in the Control ones (Fig. 3C).

To detect general trends among these carbohydrate-digesting enzymes, we performed multivariate analyses. We observed a clear separation between the evolutionary regime, colonization status, and developmental stages (Fig. 3B, left, and Table S3). However, we observed no interactions between colonization status and evolutionary regime (Fig. 3B, left, and Table S3). Furthermore, the PCA correlation circle on carbohydrate-digesting enzymes shows that amylase and maltase expression patterns are uncorrelated (Fig. 3B, right).

Looking at the pattern and activity of carbohydrate-digesting enzymes, it is hard to interpret the role of carbohydrate digestion in the context of beneficial effects of microbiota. However, the evolutionary adaptation of Selected populations may be mediated in part by enhanced carbohydrate breakdown. An increased maltose hydrolysis rate due to enhanced maltase expression would cause an increase in glucose levels, inducing the negative feedback loops (15, 16) to dampen amylase expression and activity in these flies.

### Colonization of selected and control larvae by microbiota.

**FIG 4:**
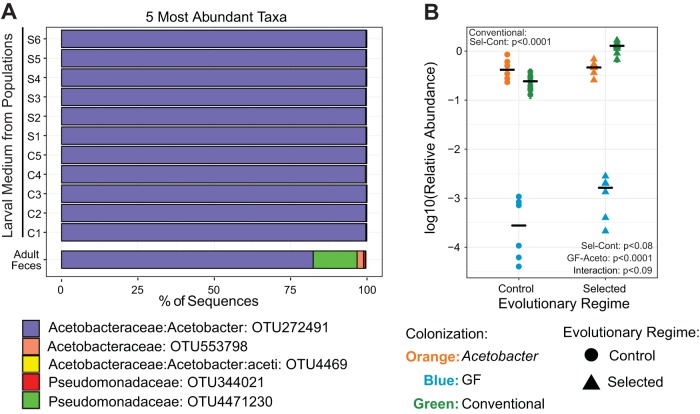
Microbiota of Selected and Control populations. (A) Identities and relative abundances of the 5 most abundant taxa in the mixed adult feces (used as the source of inoculum) and in the larval poor medium of Selected (S) and Control (C) populations previously colonized with that inoculum, assigned by 16S rRNA gene amplicon sequencing. (B) Abundance of *Acetobacteraceae* relative to the host DNA in GF, *Acetobacter*-monoassociated, and conventionally reared (as in the experimental evolution) Selected and Control populations measured by qPCR. Symbols represent means ± standard errors of the means for each population. Black bars represent the mean for the six populations within each regime. Main effect differences analyzed by GMM are represented in the panel. Interaction refers to colonization × evolutionary regime. Details are presented in Table S4.

The differential effects of microbiota on survival, development, and digestive enzyme activity of the Selected versus Control larvae reported above might result from differential colonization of their guts by the microbiota from the fecal transplant used as inoculum in these experiments. This inoculum was obtained from adult feces, and while still containing more than 80% of the dominant *Acetobacter* sp., it was somewhat more diverse (Fig. 4A, bottom bar) than the microbiota associated with the larval stage under the experimental evolution regimes, as reported above (Fig. 1A). This left the possibility that association with Control versus Selected larvae might have promoted different members of this microbial community. To check this, we collected samples of the medium at the end of larval development (this was done in the same experiment that provided larvae for the quantification of digestive enzyme expression described above). 16S rRNA gene sequencing of these samples revealed that, irrespective of the evolutionary history of the populations, they all consisted almost exclusively (>99% of the community) of the same *Acetobacter* operational taxonomic unit (OTU) already prevalent in the inoculum and under the experimental evolution conditions (Fig. 4A).

10.1128/mBio.01496-17.6 TABLE S4  Detailed statistics for data presented in Fig. 4B. Download TABLE S4, PDF file, 0.01 MB. Copyright © 2017 Erkosar et al. 2017 Erkosar et al. This content is distributed under the terms of the Creative Commons Attribution 4.0 International license.

Could then the differences in larval performance and digestion between Selected and Control populations upon microbiota association be mediated by different degrees of colonization of their guts by this dominant *Acetobacter* strain? To address this question, we monocolonized freshly hatched GF larvae of all 12 populations with this strain, allowed them to develop on the poor diet, and estimated the amount of bacteria inside the larval gut at the end of larval development. This was done by using quantitative PCR (qPCR) to quantify bacterial DNA (using primers specific to *Acetobacteraceae* 16S rRNA gene) relative to host genomic DNA (using primers for *Actin*). We found no systematic difference between these experimentally colonized Selected and Control larvae in the amount of bacterial DNA relative to host DNA (Fig. 4B, orange symbols, and Table S4), nor in the absolute threshold cycle (*C*_*T*_) values for the bacterial DNA (Fig. S1). The latter indicates that the amount of bacterial DNA in these samples was about 1,000-fold above the detection threshold; based on preliminary data (not shown), this roughly corresponds to 600 to 900 CFU per larvae. Analogous *C*_*T*_ values for GF larvae were comparable to what was observed in a mock sample containing only sterilized water, which sets the detection limit (black line in Fig. S1). This shows that our procedure of generating GF animals was effective.

10.1128/mBio.01496-17.1 FIG S1  Raw *C*_*T*_ values for *Acetobacteraceae* abundance in Selected and Control populations. *C*_*T*_ values for Selected and Control populations under different conditions are represented by dots. The horizontal line represents the *C*_*T*_ value from water control. Note that GF larvae have *C*_*T*_ values similar to the water control. Download FIG S1, PDF file, 0.01 MB. Copyright © 2017 Erkosar et al. 2017 Erkosar et al. This content is distributed under the terms of the Creative Commons Attribution 4.0 International license.

The above results indicate that Selected and Control populations become similarly colonized by the dominant *Acetobacter* strain upon experimental inoculation followed by development on the poor diet. This implies that adaptation of Selected populations to the poor diet did not cause any changes in the gut that would affect its colonization by commensals. However, this does not preclude a difference in the amount of bacteria that the populations normally harbor under their respective evolutionary regimes (in their “conventional” environment), given that the regimes differ in diet and do not involve experimental inoculation. To address this issue, we used the same approach to quantify bacterial colonization by *Acetobacter* in the third-instar larvae in the main cultures used to propagate these populations under the experimental evolution that is ongoing in the lab (i.e., on the poor diet for Selected and on the standard diet for Control populations). These larvae reared in their respective conventional environments were colonized with quantities of *Acetobacteraceae* comparable with those in the experimental inoculations (Fig. 4B, green symbols). This suggests that the ability of Selected lines to become largely independent of microbiota (i.e., their ability to cope with being GF) is a physiological result of being adapted to a poor diet and not of being maintained GF (or nearly so) as a by-product of the culture regime.

### Growth rate, transcriptome, and dFOXO target expression.

**FIG 5:**
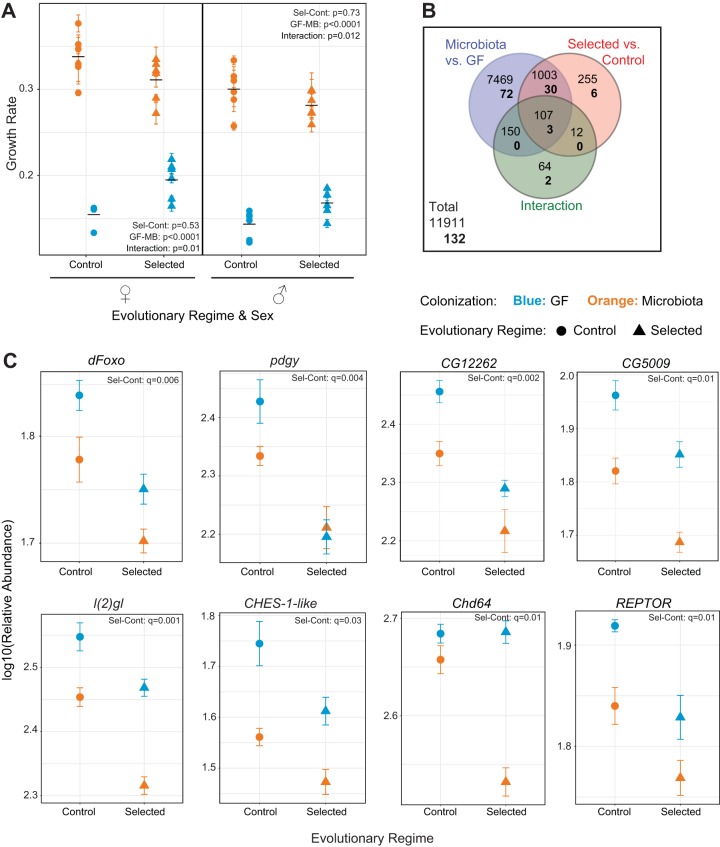
Growth rate and dFOXO targets are regulated differently by microbiota association in Selected and Control populations. (A) Growth rate on poor medium for males and females of Selected and Control populations with and without microbiota. Main effect differences analyzed by GMM are represented in the panel. Black bars represent the means for 6 populations. Detailed statistics are presented in Table S5. (B) Number of genes that are differentially expressed between GF and microbiota-associated larvae and between Selected and Control populations and those showing a statistical interaction between the two effects (i.e., where the effect of microbiota differs between the evolutionary regimes) at a 5% FDR. The top number refers to all genes; the bottom number (in bold) refers to dFOXO targets. (C) Relative transcript abundance of a biologically relevant selection of dFOXO targets obtained by transcriptome profiling (RNA-seq) from whole late-third-instar Selected and Control larvae in a GF or *Acetobacter*-associated state. Points represent means ± standard errors of the means for 6 populations. The full list of dFOXO targets with detailed statistics is represented in Table S6.

Previously, *Acetobacter pomorum* has been shown to promote larval growth on a protein-poor diet (8). Based on our findings on digestion, developmental time, and survival reported above, we expected that microbiota (containing mainly *Acetobacter*) would also strongly promote larval growth (i.e., not only fast larval development but also mass gain) in Control populations, but to a lesser degree in Selected populations. However, adult size is not a good proxy for larval growth rate in our populations: because Selected populations evolved a smaller critical size for metamorphosis initiation, they reach a smaller adult size than Controls despite growing faster on the poor diet (10, 13). Therefore, we combined adult body size (dry weight) of freshly emerged adults (Fig. S2) with developmental time data (Fig. 1A) to estimate mean larval growth rate of each population under both microbiota conditions, following the approach described in reference 10. As expected, we found that inoculation with microbiota increased larval growth rates of all populations, but this effect was significantly greater in Control than in Selected populations (as evidenced by the significant interaction between regime and bacterial treatments [Fig. 5A and Table S5]). This suggests that Selected and Control populations evolved quantitative differences in mechanisms that regulate growth in response to poor diet and microbiota association.

10.1128/mBio.01496-17.2 FIG S2  Dry adult weights of Selected and Control populations. The mean dry weight ± SEM for each population is represented by dots. Black bars represent the mean for the six replicate populations in each regime. Interaction = colonization × regime. Triple interaction = colonization × regime × sex. Adult weight is smaller in Selected than Control populations because the former have a smaller critical size (11). Download FIG S2, PDF file, 0.04 MB. Copyright © 2017 Erkosar et al. 2017 Erkosar et al. This content is distributed under the terms of the Creative Commons Attribution 4.0 International license.

10.1128/mBio.01496-17.7 TABLE S5  Detailed statistics for data presented in Fig. 5A. Download TABLE S5, PDF file, 0.02 MB. Copyright © 2017 Erkosar et al. 2017 Erkosar et al. This content is distributed under the terms of the Creative Commons Attribution 4.0 International license.

10.1128/mBio.01496-17.8 TABLE S6  Univariate analysis of differential expression of 132 dFOXO targets. Download TABLE S6, PDF file, 0.02 MB. Copyright © 2017 Erkosar et al. 2017 Erkosar et al. This content is distributed under the terms of the Creative Commons Attribution 4.0 International license.

A prime candidate for such a mechanism is the transcriptional regulation mediated by the transcription factor Forkhead box, subgroup O (dFOXO). dFOXO mediates the systemic control of larval growth in response to insulin/IGF-like (IIS) and TOR signaling, regulating ribosome biogenesis and cellular growth and proliferation (12, 17). Both IIS and TOR pathways are involved in the promotion of larval growth by microbiota (6, 8). During larval development, a large portion of the transcriptome is responsive to nutrition, and this regulation is highly dFOXO dependent: 1,250 genes in the larval body wall (containing mostly muscle) and 962 genes in the adipose tissue were reported to be dFOXO dependent (18). Moreover, dFOXO was found to bind to 521 genomic sites in the vicinity of 472 genes (18).

To address dFOXO-dependent transcriptomic changes associated with the physiological differences in Selected and Control populations, and their interaction with microbiota, we used transcriptome sequencing (RNA-seq). We obtained gene expression profiles of whole late-third-instar larvae, raised on the poor diet either under the GF condition or in monoassociation with the *Acetobacter* sp. isolated from our populations. We found 1,377 genes to be differentially expressed between Selected and Control lines at a 5% false discovery rate (FDR) and 8,729 differentially expressed between GF and *Acetobacter*-colonized larvae (Fig. 5B). Furthermore, 333 genes showed an interaction between evolutionary regime and colonization status, implying that the effect of bacteria on their expression differed between Control and Selected populations.

As the first finding supporting the involvement of dFOXO, RNA-seq revealed that the dFOXO gene itself is differentially expressed between Selected and Control populations (Fig. 5C). In its active form, dFOXO translocates to the nucleus to initiate a transcriptional response to nutrient shortage. Thus, increased levels of dFOXO should be associated with a more pronounced response to nutrient shortage. This is consistent with the higher expression of dFOXO in GF than in *Acetobacter*-colonized larvae, as well as in Control than Selected populations, given that the latter are less sensitive to nutrient shortage.

We also found that many genes known to be regulated by dFOXO were differentially expressed in response to *Acetobacter* colonization as well as between Selected and Control populations. We used a conservative candidate target list of 140 genes that were both found to be in the vicinity of dFOXO binding sites and shown to be dFOXO dependent at least in one tissue (adipose tissue or body wall) (18). Of those, 132 were present in our RNA-seq data set (eight were filtered out due to low expression levels). One hundred five of them were differentially expressed between *Acetobacter*-colonized and GF larvae at a 5% FDR (Fig. 5B). Furthermore, with 39 of these 132 genes significantly different under a 5% FDR, they were highly overrepresented among genes differentially expressed between Selected and Control populations (Fisher’s exact test, *P* < 0.0001); almost half (63 out of 132) were significantly different under the nominal (uncorrected) *P* value of <0.05 (Table S6). Importantly, of the 33 dFOXO targets significantly affected by both colonization and evolutionary regime (Fig. 5B), 31 show the same direction of change between GF and colonized states as between Control and Selected populations (Table S6). Some of these genes are involved in energy and lipid metabolism (e.g., *pdgy* [19], *CG12262* [20], and *CG5009* [21]), whereas others are involved in cell division [*l(2)gl* (22) and *CHES-1-like* (23)]. *Chd64*, which responds differently to *Acetobacter* colonization between Selected and Control populations, is involved in juvenile hormone-mediated growth regulation (24), and *REPTOR* is a major transcription factor that controls ~90% of the transcriptional regulation that occurs upon TORC1 inhibition (25) (Fig. 5C). Altogether, these results point to a differential regulation of dFOXO expression and activity in terms of downstream target activation in Selected versus Control larvae, supporting its role in mediating differences that we observe in growth rate in these populations.

We performed Gene Ontology (GO) enrichment analysis to identify the functional categories that were overrepresented among differentially expressed genes. Genes involved in proteolysis were enriched among genes that were differentially expressed between Selected and Control populations, as well as those that showed statistical interaction with the bacterial treatment (Table 1). Other enriched GO terms included mitotic cytokinesis and fatty acid beta-oxidation for both the evolutionary regime effect and *Acetobacter* colonization and DNA replication for the interaction between these two factors. Association with *Acetobacter*, irrespective of evolutionary regime, appears to affect a wide range of biological processes from cytoplasmic and mitochondrial translation to tissue morphogenesis, a sign of profound effect on the biology of the host, in accordance with other studies (26, 27).

**TABLE 1:** GO terms significantly enriched (at 5% FDR) among genes showing significantly differential expression between Selected and Control populations, between *Acetobacter*-colonized and GF larvae, or a significant statistical interaction between these two factors

Factor and term	No. of GO hits		*P* value	
	Significant^a^	Total^b^	Raw	Adjusted
Selected-control				
GO:0000281, mitotic cytokinesis	20	65	2.7E−6	4.6E−3
GO:0006635, fatty acid beta-oxidation	13	29	3.3E−6	5.7E−3
GO:0006508, proteolysis	73	475	6.9E−6	1.2E−2
All GO terms	994	10,996		
				
GF-*Acetobacter*				
GO:0032543, mitochondrial translation	82	87	5.5E−15	1.0E−11
GO:0002181, cytoplasmic translation	91	100	3.8E−14	7.1E−11
GO:0055085, transmembrane transport	218	290	2.8E−12	5.3E−9
GO:0055114, oxidation-reduction process	294	412	1.6E−11	2.9E−8
GO:0005975, carbohydrate metabolic process	88	106	5.2E−9	9.7E−6
GO:0006351, transcription, DNA templated	261	376	1.8E−8	3.3E−5
GO:0006511, ubiquitin-dependent protein catabolic process	62	71	3.5E−8	6.6E−5
GO:0008340, determination of adult lifespan	126	170	6.4E−7	1.2E−3
GO:0048813, dendrite morphogenesis	135	184	6.4E−7	1.2E−3
GO:0007476, imaginal disc-derived wing morphogenesis	155	217	1.3E−6	2.5E−3
GO:0043161, proteasome-mediated ubiquitin-dependent protein catabolic process	71	88	1.7E−6	3.1E−3
GO:0000281, mitotic cytokinesis	55	65	2.1E−6	3.9E−3
GO:0045893, positive regulation of transcription, DNA templated	91	119	3.3E−6	6.1E−3
GO:0008152, metabolic process	108	146	5.2E−6	9.8E−3
GO:0016567, protein ubiquitination	108	146	5.2E−6	9.8E−3
GO:0006099, tricarboxylic acid cycle	37	41	8.7E−6	1.6E−2
GO:0006120, mitochondrial electron transport, NADH to ubiquinone	34	37	9.9E−6	1.8E−2
GO:0006635, fatty acid beta-oxidation	28	29	1.0E−5	1.9E−2
GO:0007424, open tracheal system development	78	102	1.9E−5	3.5E−2
All GO terms	6,087	10,996		
				
Interaction				
GO:0006508, proteolysis	43	475	1.6E−13	2.4E−10
GO:0006260, DNA replication	12	51	2.6E−8	3.9E−5
All GO terms	266	10,996		

^a^ Number of genes associated with a given GO term that were found to be differently expressed at 5% FDR.

^b^ Total number of genes associated with a given GO term that were included in the transcriptome data after filtering.

## DISCUSSION

We set out to address the physiological bases of evolutionary adaptation to chronic nutritional stress, expecting that they would involve an improved ability of the animal host to exploit its microbiota. Instead, we found that our experimentally evolved Selected populations of *Drosophila* became much less dependent on microbiota for their survival and development in fewer than 200 generations of evolution on a nutrient-poor larval diet. Dependence on microbiota remained strong in our Control populations, which originated from the same base population as the Selected populations but did not have a history of evolution on the poor diet.

The dependence of *Drosophila* larvae facing nutrient shortage on benefits provided by gut microbiota is known to be mediated at least in part by two major mechanisms: enhanced digestion and induction of IIS and TOR signaling, which regulate cell and tissue growth via the transcription factor dFOXO (6–8). We show that both digestion and dFOXO-dependent transcription became modified in our Selected populations genetically adapted to a poor diet. These changes in part mimic the effects of microbiota, providing a likely explanation for the reduced dependence of Selected larvae on microbiota. Our results from the Control populations also provide new insights into the effects of microbiota on larvae from populations not adapted to poor diets.

### Adaptation to poor diet affects digestion in a microbiota-dependent way.

Animal digestive systems never manage to extract all nutrients from the ingested food, and increasing the fraction that is extracted (digestive efficiency) is one way in which the consequences of a nutrient-poor diet can be alleviated (28). Digestion-mediated benefits on larval growth are well documented for a common member of the *Drosophila* microbiota, *Lactobacillus plantarum*. This commensal induces transcription of a set of digestive enzymes, in particular Jonah proteases, leading to enhanced proteolytic activity in the gut and a higher amino acid/dipeptide uptake in the body (7). Our data from the Control populations extend these results by showing that this effect is not specific to lactobacilli but also occurs in association with *Acetobacter*. They also reveal that, even though they are all serine proteases, trypsins and Jonah proteases respond differently to *Acetobacter* colonization, indicating that these two sets of proteases may be functionally different. Furthermore, we find that association with microbiota suppresses amylase expression and activity, further underlining that the digestive response to microbiota is a complex response rather than a general upregulation of all classes of digestive enzymes. In the GF state, the Selected larvae show a higher proteolytic activity (at least in mid-third larval stage) and lower amylase activity than GF Controls, which parallels the effects of microbiota on Control larvae. This is not the case for maltase expression. Although in the GF state Selected larvae produce larger amounts of maltases, which would correlate with higher maltose breakdown in these larvae, addition of microbiota does not systematically induce maltase expression. However, we cannot exclude the possibility that carbohydrate metabolism in bacteria also influences the amount of monosaccharides present in the system and thus the feedback loops that control the activity and expression of these enzymes.

Interestingly, protease and amylase activity of Selected larvae is less strongly affected by microbiota than is the case for Control larvae. This correlates with the growth and survival pattern that we observe. Selected populations that are able to grow and survive already well with their basal (i.e., germ-free) digestive efficiency are less sensitive to microbiota than Control populations, which need to enhance their digestion to sustain growth.

### Evolutionary changes in dFOXO-dependent gene expression mimic the effects of microbiota.

The transcription factor dFOXO (like its homologs in other animals) is a key effector in responses to systemic nutrient shortage: its activation in response to low IIS and TOR signaling mediates a transcriptional response that suppresses larval growth (12, 17). Microbiota (both *Lactobacillus* and *Acetobacter*) are known to induce IIS and TOR signaling pathways in *Drosophila* larvae growing on poor diets, reducing dFOXO-dependent suppression of cell and tissue growth (6, 8). Consistent with this, we show that the expression of most dFOXO targets changes in response to *Acetobacter* colonization. Furthermore, in the course of experimental evolution of Selected populations on the poor diet, the expression of many dFOXO targets changed in a way that mimics the effects of microbiota. This suggests that evolution in response to nutrient shortage acted in part by releasing the larvae from dFOXO-mediated growth suppression, modifying some of the same mechanisms that mediate responses to microbiota in nonadapted larvae.

We also find that the expression of dFOXO itself is downregulated by association with microbiota as well as in Selected compared to Control populations. Although FOXO-dependent regulation is known to occur mainly through posttranslational modifications which control its translocation from the cytoplasm to the nucleus (29), the expression of a mammalian FOXO gene has been reported to increase in response to nutrient shortage in rats (30). Thus, changes in dFOXO expression may be a complementary way in which FOXO-dependent responses to environmental factors and genetically based evolutionary changes could be modulated.

### Reduced dependence on microbiota as a side effect of adaptation to poor diet.

The differences between Selected and Control populations have been produced by fewer than 200 generations (10 years) of experimental evolution under different dietary regimes. The fact that the changes in digestion and dFOXO-dependent transmission evolved in a repeatable way across six independent Selected populations implies that these changes improved Darwinian fitness under the poor diet regime (9). Thus, although other traits may also have contributed to the adaptation phenotype evolved during the 10 years of experimental evolution, changes in digestion and FOXO-dependent transcription explain at least in part how Selected populations adapted to poor diet and became less dependent on microbiota.

The reduced dependence of our Selected populations on microbiota for larval growth and survival under strong nutrient limitations is intriguing from an evolutionary viewpoint. Our quantification of microbiota implies that under their culture regime Selected populations are exposed to microbiota similarly to Control populations and become colonized by a quantity of bacteria comparable to that resulting from our experimental inoculations. This suggests that the reduced dependence of Selected populations on microbiota is not a consequence of being underexposed to bacteria in the course of their experimental evolution but a direct effect of adaptation to nutrient shortage under the strong selection imposed by the extremely poor diet. Possibly, in spite of the positive net effect of microbiota on Control larvae, the microbiota may have competed with the host for nutrition on the poor diet, which may have acted as a selective force for adaptation on the Selected populations to increase nutrient efficiency. As we have reported elsewhere (13), the Selected populations also evolved a greater susceptibility to the Gram-negative intestinal pathogen *Pseudomonas entomophila*. Thus, evolutionary adaptation to nutritional stress may affect interactions between the host and both beneficial and harmful gut microbes. Given that the relationship between animals and gut microbiota likely goes back hundreds of millions of years, it is remarkable that fruit flies retained the potential to rapidly evolve a markedly reduced dependence on gut microbiota for fitness under nutritional stress.

## MATERIALS AND METHODS

### Experimentally evolved fly populations and diet.

Six replicate Selected and six replicate Control populations were maintained at 20°C and 70% humidity, with a 12-/12-h dark/light cycle on a 21-day generation cycle. Control populations were cultured on standard cornmeal (5%)-yeast (1.25%)-sugar (3% sucrose, 6% glucose) medium, and Selected populations were cultured on poor medium containing one-fourth of the nutrients during larval development (10). Experimental evolution was carried out as described in detail in reference 10. All 12 populations originated from the same base population. At each generation, eggs were collected on live yeast, leading to contamination of egg surfaces with yeast, which may cause alterations in the gut microbiota of larvae. Eggs were rinsed with tap water to enable egg counting, which dilutes the flies’ natural microbiota and causes environmental contamination. Approximately 200 eggs were collected from adults of each population and distributed on their respective media for larval growth. Upon emergence, adults from all populations were transferred to standard medium supplemented with dry yeast. Experiments were carried out between generation 177 and generation 200. Before each experiment, populations were reared on standard medium for >2 generations to avoid maternal effects.

To avoid changes in the conventional recipe, we kept the food clean by boiling it. The food used in the experiments was boiled for >10 min and poured at 78°C in autoclaved fly bottles using tools sterilized with 70% ethanol under the hood.

### Preparation of gnotobiotic larvae.

Embryos were collected from an overnight egg laying on orange juice-agar plates supplemented with yeast. Embryos were washed with tap water, sterilized by soaking in 5% bleach for 3 min, and rinsed with autoclaved water. Two hundred eggs were counted on a mesh, under a stereomicroscope, next to a Bunsen burner to avoid further contamination. Counted eggs were transferred to fly bottles containing standard or poor food medium.

For the GF treatment, 300 µl of heat-inactivated bacteria (developmental time experiment) or sterile phosphate-buffered saline (PBS) (enzymatic activity assays, qRT-PCR, and RNA-seq experiments) was added on the sterile embryos.

To colonize larvae with microbiota, fecal transplantation was used. Adults (10 males and 10 females) were collected from all populations and kept on standard food for 5 days. They were transferred on a petri dish with a slice of medium and allowed to defecate for 48 h. Feces were collected after removal of the medium using an ethanol-washed brush in sterile PBS. Feces were filtered through a previously bleached and rinsed mesh, and remaining suspension was adjusted to a culture turbidity (optical density [OD]) of 1 to have approximately 10^9^ cells. Three hundred microliters was inoculated on the embryos for colonization.

To monoassociate larvae with *Acetobacter*, bacteria were grown for 48 h at 30°C under agitation in de Man, Rogosa, and Sharpe (MRS) medium (Difco; catalog no. 288110) supplemented with 2.5% d-mannitol (Sigma; catalog no. M1902). Bacteria were harvested by centrifugation at 3,000 rpm for 10 min and diluted with sterile PBS to reach an OD of 1. Three hundred microliters of culture was added on sterile embryos.

### Developmental time and survival.

To measure developmental time and egg-to-pupa survival, gnotobiotic animals were prepared as described above. For each population and condition (GF versus microbiota-colonized), three replicate larval culture bottles were set up with 200 embryos per 30 ml of the poor diet medium (72 bottles in total). Newly formed pupae were scored every day to determine larval development time and the proportion of larvae surviving to pupation.

### Adult dry weight and growth rate.

The first group of adults emerging from poor diet was discarded on the day of emergence, and newly emerging ones were collected within 48 h of eclosion. Ten males and 10 females from each bottle were picked randomly, separated, and frozen at −20°C. When the number of adults was not sufficient (valid for GF Control populations), the procedure was repeated, and adults emerging on different days were pooled. If the number of adults was less than 10, the sample was discarded. To determine the dry body weight, flies were dried at 80°C for 2 days and weighed on a precision balance.

According to the procedure in reference 10, larval growth rate on poor diet was estimated separately for each sex and population as ln(final size/initial size)/(time available for growth). Final size was the mean dry weight of adults, and initial size was assumed to be 0.005 mg, the approximate dry weight of an egg (10). Time available for growth was estimated as the egg-to-adult time minus 48 h to account for the time needed for egg hatching, the fact that pupae were scored at 24-h intervals, and the time that the larvae spend wandering before pupation (which does not differ between the Selected and Control populations [31]). While this estimate is necessarily approximate, all conclusions about growth rate were robust to changing the time available for growth by ±24 h.

### Nucleic acid extraction and qPCR.

RNA extractions were performed from three biological replicates of 10 dissected midguts or 10 whole larvae from all six Selected and six Control populations (resulting in 72 gut samples for each time point and 72 whole larval RNA samples) using the RNeasy minikit (Qiagen). Reverse transcription was performed as described in reference 7.

DNA extraction was carried out from samples containing 10 surface-sterilized (upon washing in sterile water and ethyl alcohol [EtOH]) larvae using the DNeasy Blood and Tissue kit (Qiagen) according to the manufacturer’s protocol adapted for insect cells. Specific collection times (days after egg laying) for each condition were as follows: control GF, 19; Control *Acetobacter*, 10; Selected GF, 13; Selected *Acetobacter*, 10; Control conventional, 5; and Selected conventional, 6. For conventionally reared lines, larvae were collected from two replicate vials per population. Monoassociated and GF groups were collected from one vial per population.

qPCR was carried out using gene-specific primer sets (available as supplemental material in reference 7 or upon request), using the Power SYBR green PCR master mix (Life Technologies; catalog no. 4368702) under the following conditions: 95°C for 10 min and 40 cycles of 95°C for 15 s and 60°C for 1 min. Melting curve analysis ensured amplification of a single product. Ratios of gene of interest to reference gene (2^−Δ*CT*^) were log transformed for statistical analysis.

### Protease activity assay.

Twenty to 50 whole larvae (equivalent to a volume of 40 µl) were collected from 6 Selected and 6 Control populations in three biological replicates at different time points, resulting in 198 individual samples to process. Larvae were collected from the beginning of third instar until the emergence of first pupa. Wandering larvae at the last time point were excluded. Specific collection times (days after egg laying) for each condition were as follows: control GF, 8, 13, 17, and 19; Control microbiota-colonized (MB), 7 and 11; Selected GF, 7, 11, and 16; and Selected MB, 6 and 11. Protease activity was measured using an azocasein assay as described in reference 7, which was optimized for whole larvae.

### Amylase activity assay.

Amylase activity was measured using the amylase activity assay kit (Sigma; catalog no. MAK009) according to the manufacturer’s instructions and using the same samples as in the protease activity assay. Fifty microliters of sample was added to the substrate mix on a 96-well plate. Absorbance at 405 nm was read every 20 min for 17 h at 25°C. The rate of the reaction, the *k* constant, was calculated using nonlinear least-squares (nls) models in R using function *wrapnls* in package *nlmrt* with the equation *y = c* + *A* (*1* − *e*^*−kt*^). The rate was normalized to total protein quantity as for the protease activity assay.

### 16S rRNA gene sequencing.

Community profiling was from whole larvae, adult feces, and poor-medium-colonizing bacteria during larval stages. Ten whole larvae were collected at late L3 stage, rinsed in sterile water, and surface sterilized with 70% EtOH. Adult feces collection is described under “Developmental time and survival” above. Larval medium was washed with 10 ml sterile PBS. The resulting solution was centrifuged for 1 min at 3,000 rpm to precipitate the food. The supernatant was recentrifuged at 13,000 rpm for 10 min. Bacterial pellet was resuspended in 1 ml sterile PBS. Five microliters of this suspension was used directly in the PCR to amplify the V1-V2 regions of the 16S rRNA gene, without any DNA extraction. Regions were amplified using the Kapa HiFi HotStart ReadyMix (Kapa Biosystems; catalog no. KK2601) and primers 8-27F (5′ TCGTCGGCAGCGTCAGATGTGTATAAGAGACAGAGAGTTTGATCMTGGCTCAG 3′) and 339-356R (5′ GTCTCGTGGGCTCGGAGATGTGTATAAGAGACAGTGCTGCCTCCCGTAGGAG 3′) including adapter sequences (underlined) for the second PCR round. Three replicate 25-µl PCR mixtures containing 10 ng µl^−1^ DNA and 1 µM (each) primer were carried out under the following conditions: 95°C for 3 min and 25 cycles of 95°C for 30 s, 56°C for 15 s, and 72°C for 30 s, followed by a final incubation at 72°C for 5 min. Products were pooled from triplicate reaction mixtures and verified for amplicon size on a Fragment Analyzer (Advanced Analytical Technologies, Inc.). Libraries were prepared and sequenced at the Lausanne Genome Technology Facilities of the University of Lausanne according to the Illumina 16S metagenomic sequencing library preparation protocol. Briefly, first-round PCR products were cleaned up using AMPure XB (Beckman Coulter Genomics; catalog no. A63881) beads. An index PCR was carried out on the purified fraction using a Nextera XT index kit (Illumina; catalog no. FC-131-1001) to produce sequencing libraries. Libraries were again verified with a Fragment Analyzer, mixed with 20% PhiX library (Illumina; catalog no. FC-110-3001), and subjected to Illumina MiSeq paired-end sequencing in one lane, with all libraries multiplexed.

All steps of sequence analysis were performed using the QIIME 1.8.0 bioinformatics software (32). Raw 300-bp paired-end reads were filtered by size (minimum 100-bp overlap between paired ends) and quality (Phred scores of ≥30). Chimeric reads were eliminated using the Usearch algorithm (33, 34). Reads were classified into operational taxonomic units (OTUs) using the open reference OTU clustering pipeline, excluding the prefiltering step and using the *uclust* method (33). Reads were aligned to the Greengenes database (35) using PyNAST (36) with a 99% identity threshold, to have specificity down to the species level. Taxonomies were assigned using the RDP classifier (37), and phylogenetic trees were built using FastTree 2.1.3 (38). Reads matching endosymbiont *Wolbachia* were excluded from the analysis.

### Isolation of *Acetobacter* sp.

To isolate *Acetobacter*, media from one Control and one Selected population (randomly chosen) were streaked on MRS-mannitol plates. A single colony was used to prepare liquid cultures (as described under “Preparation of gnotobiotic larvae”) and to establish glycerol stocks, as well as for 16S rRNA gene full-length amplification using universal primers (sequences available upon request) and Kapa HiFi HotStart ReadyMix. The 16S rRNA gene product was sequenced using Sanger sequencing (GATC Biotech). The obtained sequence was assigned to *Acetobacter* using RDP classifier (https://rdp.cme.msu.edu/classifier/classifier.jsp). To ensure that we isolated the dominant strain, which was detected during community profiling, we aligned sequences using APE software.

### Statistical analysis.

Univariate analysis was performed using general linear mixed models (GMM) using Satterthwaite approximation for the degrees of freedom (Proc Mixed of SAS v.9.3). Multivariate analysis was done using the “ade4” package in R (39). Evolutionary regime (Selected or Control) and microbiota treatment (germ-free or colonized) were fixed factors; time point was also a fixed factor except for enzyme activity assays, where more than two time points were included. Replicate populations were treated as a random factor nested in evolutionary regimes. *A priori* pairwise contrasts were performed within the framework of the GMM (using the Slices option of Proc Mixed). The detailed output of all analyses can be found in Tables S1 to S5 in the supplemental material.

### RNA-seq analysis.

Libraries were generated using the TruSeq Stranded Total RNA Library Prep kit (RS-122-2201/RS-122-2202) and sequenced in two lanes, with all libraries multiplexed, on a HiSeq 2500 sequencer using single-end chemistry. The reads generated were mapped as described previously (40) using *Drosophila melanogaster* genome version BDGP6.

This yielded between roughly 15 and 24 million mapped reads assigned uniquely to genomic features (genes) for each sample. Genes having 1 count per million in at least 6 samples were retained for further analysis. Counts were then normalized for library size in the “edgeR” package (41, 42). We ran a general linear model with a regime × colonization factorial design using the “Limma” package (43) to determine which genes’ expression was significantly affected by these two factors and their interaction. We performed a GO term analysis on resulting significantly different gene lists (cutoff FDR, 5%) using DAVID (44, 45).

### Data accessibility.

Sequence data have been deposited at the NCBI Sequence Read Archive (16S rRNA gene reads, BioProject PRJNA412702, accession numbers SAMN07723061-SAMN07723084; RNAseq, BioProject PRJNA412704, accession numbers SAMN07723150-SAMN07723173). Phenotype and qPCR data have been deposited at Dryad Digital Repository (doi:10.5061/dryad.td3r1).
