# Supplementary material for: Adaptation to Chronic Nutritional Stress Leads to Reduced Dependence on Microbiota in Drosophila melanogaster
Source: mBio. 2017 Oct 24;8(5):e01496-17. doi: 10.1128/mBio.01496-17 (PMC5654931; doi:10.1128/mBio.01496-17)
Supplement: TABLE S1 [file mbo005173542st1.pdf]

**Supplemental Table S1:** Type 3 F-tests for fixed effects in general mixed model on developmental time and survival reported in Fig. 1B,C.

**Development Time**

| Effect | Num. df | Den. df | *F* | *p* |
|---|---|---|---|---|
| Evolutionary regime | 1 | 20 | 36.87 | <.0001 |
| Colonization | 1 | 20 | 258.67 | <.0001 |
| Evolutionary regime x Colonization | 1 | 20 | 29.65 | <.0001 |
| Pairwise Contrasts: | | | | |
| Selected vs Control in MB state | 1 | 16.8 | 2.38 | 0.14 |
| Selected vs Control in GF state | 1 | 16.8 | 20.1 | 0.0003 |
| MB vs GF in Control populations | 1 | 10 | 78.69 | <.0001 |
| MB vs GF in Selected populations | 1 | 10 | 24.42 | 0.0006 |

**Survival**

| Effect | Num. DF | Den. df | *F* | *p* |
|---|---|---|---|---|
| Evolutionary regime | 1 | 20 | 46.32 | <.0001 |
| Colonization | 1 | 20 | 23.58 | <.0001 |
| Evolutionary regime x Colonization | 1 | 20 | 11.78 | 0.0026 |
| Pairwise Contrasts: | | | | |
| Selected vs Control in MB state | 1 | 20 | 5.69 | 0.027 |
| Selected vs Control in GF state | 1 | 20 | 52.41 | <.0001 |
| MB vs GF in Control populations | 1 | 20 | 34.35 | <.0001 |
| MB vs GF in Selected populations | 1 | 20 | 1.01 | 0.33 |
