# Supplementary material for: Adaptation to Chronic Nutritional Stress Leads to Reduced Dependence on Microbiota in Drosophila melanogaster
Source: mBio. 2017 Oct 24;8(5):e01496-17. doi: 10.1128/mBio.01496-17 (PMC5654931; doi:10.1128/mBio.01496-17)
Supplement: TABLE S2 [file mbo005173542st2.pdf]

Supplemental Table S2. Analysis of results presented in Fig 2A,C.

(A) Multivariate analysis of variance on the expression of digestive proteases reported in Fig 2A.

Effect	Wilks λ	Num df	Den df	*F*	*p*
Regime	0.26	11	30	7.8	<.0001
Colonization	0.09	11	30	26.4	<.0001
Stage	0.12	11	30	19.9	<.0001
Regime x Colonization	0.69	11	30	1.2	0.3028
Regime x Stage	0.75	11	30	0.9	0.5321
Colonization x Stage	0.23	11	30	9.0	<.0001
Regime x Colonization x Stage	0.80	11	30	0.7	0.7505

(B) Univariate analyses of the expression of proteases reported in Fig. 2A.

Jon66Ci

Effect	*Num df*	*Den df*	*F*	*p*
Regime	1	10	0.0	0.8771
Colonization	1	10	298.8	<.0001
Regime x Colonization	1	10	2.0	0.1922
Stage	1	10	50.1	<.0001
Regime x Stage	1	10	0.2	0.6637
Colonization x Stage	1	10	49.2	<.0001
Regime x Colonization x Stage	1	10	0.2	0.688

Jon66Cii

Effect	*Num df*	*Den df*	*F*	*p*
Regime	1	10	5.4	0.0427
Colonization	1	10	160.0	<.0001
Regime x Colonization	1	10	0.0	0.948
Stage	1	10	87.1	<.0001
Regime x Stage	1	10	4.8	0.0526
Colonization x Stage	1	106	29.8	<.0001
Regime x Colonization x Stage	1	106	5.0	0.0271

Jon99Ci

Effect	*Num df*	*Den df*	*F*	*p*
Regime	1	10	2.9	0.1196
Colonization	1	10	99.2	<.0001
Regime x Colonization	1	10	0.1	0.7733
Stage	1	10	0.1	0.7876
Regime x Stage	1	10	1.2	0.296
Colonization x Stage	1	10	12.1	0.006
Regime x Colonization x Stage	1	10	3.8	0.0801

Jon44E

Effect	*Num df*	*Den df*	*F*	*p*
Regime	1	10	0.3	0.5778
Colonization	1	10	186.3	<.0001
Regime x Colonization	1	10	1.4	0.2658
Stage	1	10	1.9	0.1994
Regime x Stage	1	10	0.0	0.8656
Colonization x Stage	1	106	13.6	0.0004
Regime x Colonization x Stage	1	106	5.4	0.0219

Jon65Ai

Effect	*Num df*	*Den df*	*F*	*p*
Regime	1	10	0.2	0.6732
Colonization	1	20	570.4	<.0001
Regime x Colonization	1	20	3.1	0.096
Stage	1	10	9.0	0.0134
Regime x Stage	1	10	0.0	0.8952
Colonization x Stage	1	20	44.6	<.0001
Regime x Colonization x Stage	1	20	0.2	0.642

CG8299

Effect	*Num df*	*Den df*	*F*	*p*
Regime	1	10	9.1	0.0131
Colonization	1	10	41.2	<.0001
Regime x Colonization	1	10	0.1	0.7245
Stage	1	10	18.6	0.0015
Regime x Stage	1	10	0.9	0.379
Colonization x Stage	1	10	41.0	<.0001
Regime x Colonization x Stage	1	10	0.2	0.6971

CG18180

Effect	*Num df*	*Den df*	*F*	*p*
Regime	1	12.1	11.4	0.0055
Colonization	1	14.8	235.1	<.0001
Regime x Colonization	1	14.8	0.3	0.5829
Stage	1	16.1	20.4	0.0003
Regime x Stage	1	16.1	0.2	0.6806
Colonization x Stage	1	9.96	50.5	<.0001
Regime x Colonization x Stage	1	9.96	0.0	0.8548

CG18179

Effect	*Num df*	*Den df*	*F*	*p*
Regime	1	10	1.7	0.2175
Colonization	1	10	26.8	0.0004
Regime x Colonization	1	10	2.9	0.1216
Stage	1	10	63.6	<.0001
Regime x Stage	1	10	0.2	0.6957
Colonization x Stage	1	10	0.0	0.842
Regime x Colonization x Stage	1	10	2.8	0.1277

α-Try

Effect	*Num df*	*Den df*	*F*	*p*
Regime	1	10	2.9	0.1213
Colonization	1	116	46.1	<.0001
Regime x Colonization	1	116	1.3	0.2552
Stage	1	10	13.7	0.0041
Regime x Stage	1	10	2.4	0.1561
Colonization x Stage	1	116	15.9	0.0001
Regime x Colonization x Stage	1	116	3.3	0.0731

β-Try

Effect	*Num df*	*Den df*	*F*	*p*
Regime	1	10	2.5	0.1463
Colonization	1	20	74.8	<.0001
Regime x Colonization	1	20	5.5	0.0299
Stage	1	10	22.9	0.0007
Regime x Stage	1	10	1.3	0.2771
Colonization x Stage	1	20	16.6	0.0006
Regime x Colonization x Stage	1	20	2.8	0.1089

ξ-Try

Effect	*Num df*	*Den df*	*F*	*p*
Regime	1	10	2.5	0.1482
Colonization	1	9.48	7.2	0.0243
Regime x Colonization	1	9.48	0.4	0.5355
Stage	1	19.1	8.4	0.0094
Regime x Stage	1	19.1	0.4	0.5147
Colonization x Stage	1	19.1	1.5	0.2383
Regime x Colonization x Stage	1	19.1	1.2	0.2921

(C) General mixed model analysis of variance for the protease activity reported in Fig. 2C

Protease Activity

Effect	*Num df*	*Den df*	*F*	*P*
Regime	1	10	0.02	0.8836
Colonization	1	10.3	74.6	<.0001
Regime x Colonization	1	10.3	12.4	0.0053
Time	1	172	229.9	<.0001
Pairwise Contrasts:				
MB vs GF in Control populations	1	9.7	76.3	<.0001
MB vs GF in Selected populations	1	10.9	12.7	0.0045
Selected vs Control in GF state	1	14.3	3.5	0.0829
Selected vs Control in MB state	1	18.7	2.2	0.1541
