# Supplementary material for: Adaptation to Chronic Nutritional Stress Leads to Reduced Dependence on Microbiota in Drosophila melanogaster
Source: mBio. 2017 Oct 24;8(5):e01496-17. doi: 10.1128/mBio.01496-17 (PMC5654931; doi:10.1128/mBio.01496-17)
Supplement: TABLE S3 [file mbo005173542st3.pdf]

Supplemental Table S3. Analysis of results presented in Fig 3A,C.

(A) Multivariate analysis of variance on the expression of carbohydrate digestive enzymes reported in Fig 3C.

Effect	Wilks λ	Num df	Den df	*F*	*p*
Regime	0.28	6	35	7.8	<.0001
Colonization	0.39	6	35	26.4	<.0001
Stage	0.31	6	35	19.9	<.0001
Regime x Colonization	0.84	6	35	1.2	0.3830
Regime x Stage	0.72	6	35	0.9	0.0543
Colonization x Stage	0.79	6	35	9.0	0.1830
Regime x Colonization x Stage	0.88	6	35	0.7	0.5560

(B) Univariate analyses of the expression of proteases reported in Fig. 3C.

Amy-D

Effect	*Num df*	*Den df*	*F*	*p*
Regime	1	10	0.4	0.5367
Colonization	1	10	5.6	0.0391
Regime x Colonization	1	10	0.0	0.946
Stage	1	20	23.4	0.0001
Regime x Stage	1	20	3.9	0.0627
Colonization x Stage	1	20	5.5	0.0299
Regime x Colonization x Stage	1	20	0.2	0.682

Amy-P

Effect	*Num df*	*Den df*	*F*	*p*
Regime	1	10	4.0	0.073
Colonization	1	10	25.2	0.0005
Regime x Colonization	1	10	0.1	0.7195
Stage	1	10	68.4	<.0001
Regime x Stage	1	10	2.4	0.1504
Colonization x Stage	1	10	8.9	0.0138
Regime x Colonization x Stage	1	10	4.7	0.0556

Mal-A1

Effect	*Num df*	*Den df*	*F*	*p*
Regime	1	10	9.0	0.0133
Colonization	1	20	84.7	<.0001
Regime x Colonization	1	20	19.9	0.0002
Stage	1	10	2.4	0.1498
Regime x Stage	1	10	2.3	0.1595
Colonization x Stage	1	20	2.3	0.1478
Regime x Colonization x Stage	1	20	17.9	0.0004

Mal-A3

Effect	*Num df*	*Den df*	*F*	*p*
Regime	1	10	37.0	0.0001
Colonization	1	10	20.4	0.0011
Regime x Colonization	1	10	5.9	0.0353
Stage	1	20	88.3	<.0001
Regime x Stage	1	20	0.1	0.734
Colonization x Stage	1	20	10.1	0.0047
Regime x Colonization x Stage	1	20	2.5	0.1275

Mal-A4

Effect	*Num df*	*Den df*	*F*	*p*
Regime	1	20	10.7	0.0038
Colonization	1	20	0.2	0.6845
Regime x Colonization	1	20	0.9	0.3486
Stage	1	20	6.0	0.0242
Regime x Stage	1	20	2.5	0.1327
Colonization x Stage	1	20	3.9	0.0633
Regime x Colonization x Stage	1	20	0.9	0.3564

Mal-A8

Effect	*Num df*	*Den df*	*F*	*p*
Regime	1	10	3.0	0.116
Colonization	1	10	14.5	0.0035
Regime x Colonization	1	10	13.7	0.0041
Stage	1	10	3.4	0.0936
Regime x Stage	1	10	1.3	0.2833
Colonization x Stage	1	10	0.4	0.5525
Regime x Colonization x Stage	1	10	5.4	0.0431

(C) General mixed model analysis of variance for the amylase activity reported in Fig. 2C

Effect	*Num DF*	*Den df*	*F*	*p*
Regime	1	10	4.8	0.0533
Colonization	1	70.5	7.6	0.0074
Regime x Colonization	1	10.1	6.5	0.0284
Time	1	169	4.9	0.0282
Time x Colonization	1	168	47.8	<.0001
Pairwise Contrasts:				
MB vs GF in Control populations	1	9.4	28.1	0.0004
MB vs GF in Selected populations	1	10.8	2.5	0.1446
Selected vs Control in GF state	1	12	9.6	0.0093
Selected vs Control in MB state	1	14.4	1.0	0.3417
