# Supplementary material for: Adaptation to Chronic Nutritional Stress Leads to Reduced Dependence on Microbiota in Drosophila melanogaster
Source: mBio. 2017 Oct 24;8(5):e01496-17. doi: 10.1128/mBio.01496-17 (PMC5654931; doi:10.1128/mBio.01496-17)
Supplement: TABLE S4 [file mbo005173542st4.pdf]

Supplemental Table S4. Analysis of the relative abundance of *Acetobacter* in Selected and Control larvae experimentally inoculated with *Acetobacter* or in experimental GF state (orange and green symbols in Fig. 4B)[1].

Effect	*Num. df*	*Den. df*	*F*	*p*
Regime	1	10	3.8	0.0781
Colonization	1	10	313.9	<.0001
Regime x Colonization	1	10	3.6	0.0875
Pairwise contrast:				
Control vs Selected in *Acetobacter*-colonized state	1	20	0.01	0.9423

[1]The abundance of *Acetobacter* in conventionally rearead larvae (green symbols in Fig. 4B) has not been included in the analysis because has been done at a different time and it involves poor diet for Selected larvae but the standard diet for the Control populations, resulting in large differences in timing of sampling and body size of larvae.
