# Supplementary material for: Adaptation to Chronic Nutritional Stress Leads to Reduced Dependence on Microbiota in Drosophila melanogaster
Source: mBio. 2017 Oct 24;8(5):e01496-17. doi: 10.1128/mBio.01496-17 (PMC5654931; doi:10.1128/mBio.01496-17)
Supplement: FIG S1 [file mbo005173542sf1.pdf]

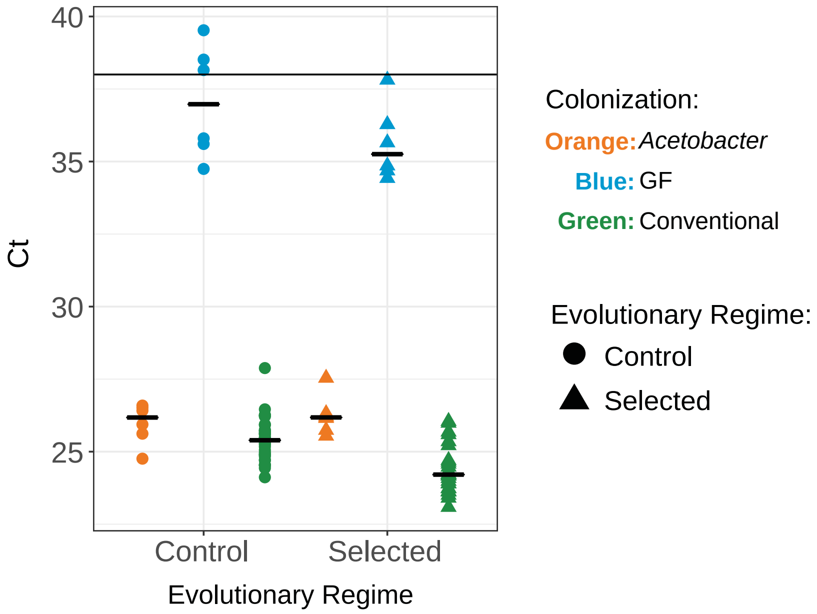

Supplemental Figure S1. Raw Ct values for Acetobacteraceae abundance in Selected and Control populations. Ct values for Selected and Control populations in different conditions are represented by dots. Horizontal line represents the Ct value from water control. Note that GF larvae have Ct values similar to the water control.
