# Supplementary material for: Adaptation to Chronic Nutritional Stress Leads to Reduced Dependence on Microbiota in Drosophila melanogaster
Source: mBio. 2017 Oct 24;8(5):e01496-17. doi: 10.1128/mBio.01496-17 (PMC5654931; doi:10.1128/mBio.01496-17)
Supplement: FIG S2 [file mbo005173542sf2.pdf]

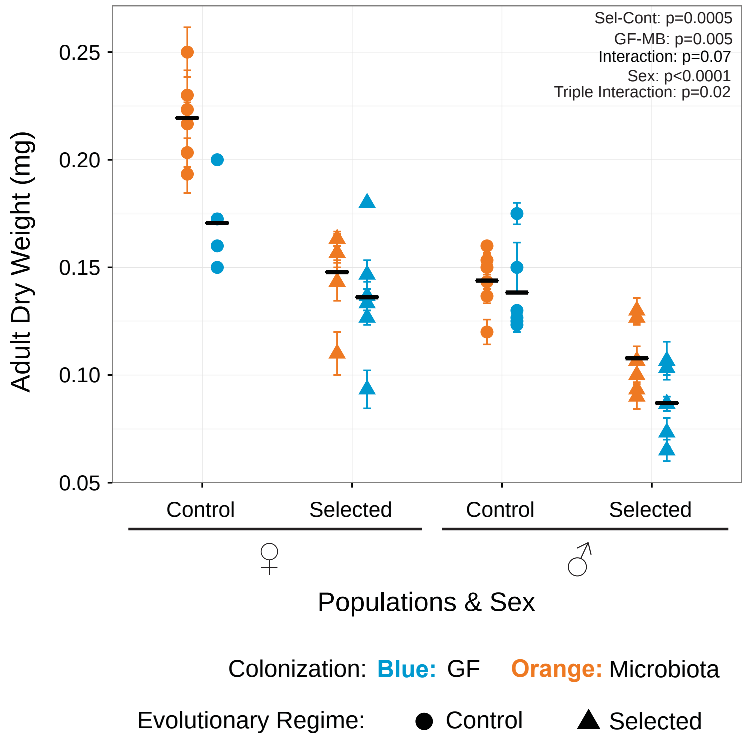

Supplemental Figure S2. Dry adult weights of Selected and Control populations. Mean±SEM dry weight for each population is represented by dots. Black bars represent the mean for the six replicate populations in each regime. Interaction = Colonization Regime. Triple Interaction = Colonization × Regime × Sex. Adult weight is smaller in Selected than Control populations because the former have a smaller critical size (11).
