# Supplementary material for: Adaptation to Chronic Nutritional Stress Leads to Reduced Dependence on Microbiota in Drosophila melanogaster
Source: mBio. 2017 Oct 24;8(5):e01496-17. doi: 10.1128/mBio.01496-17 (PMC5654931; doi:10.1128/mBio.01496-17)
Supplement: TABLE S5 [file mbo005173542st5.pdf]

Supplemental Table S5. Tests of fixed effects in the general mixed model analysis of growth rate (results presented in Fig. 5A).

Growth rate, females

Effect	*Num df*	*Den df*	*F*	*p*
regime	1	9.2	0.4	0.5303
bacteria	1	8.8	213.2	<.0001
regime*bacteria	1	8.8	10.8	0.0097
Pairwise contrasts:				
MB vs GF in Control populations	1	10.5	138.1	<.0001
MB vs GF in Selected populations	1	6.9	76.1	<.0001
Selected vs Control in GF state	1	21	6.6	0.0177
Selected vs Control in MB state	1	17.7	4.0	0.0615

Growth rate, males

Effect	*Num df*	*Den df*	*F*	*p*
regime	1	20.5	0.1	0.7256
bacteria	1	20.5	300.2	<.0001
regime*bacteria	1	20.5	7.6	0.0118
Pairwise contrasts:				
MB vs GF in Control populations	1	21.6	195.0	<.0001
MB vs GF in Selected populations	1	19.3	109.8	<.0001
Selected vs Control in GF state	1	21.6	4.7	0.0414
Selected vs Control in MB state	1	19.3	3.0	0.0988
