# Supplementary material for: Adaptation to Chronic Nutritional Stress Leads to Reduced Dependence on Microbiota in Drosophila melanogaster
Source: mBio. 2017 Oct 24;8(5):e01496-17. doi: 10.1128/mBio.01496-17 (PMC5654931; doi:10.1128/mBio.01496-17)
Supplement: TABLE S6 [file mbo005173542st6.pdf]

Supplemental Table S6. Univariate analysis of differential expression of 132 dFOXO targets: estimated $\log_2$ fold-change (logFC), uncorrected (raw) *p*-value and adjusted *p*-value (i.e., *q*-value) corresponding to the main effect of selection regime (Selected – Control populations) and the colonization treatment (*Acetobacter* – GF).

FB Gene ID	Symbol	Selected – Control			*Acetobacter* – GF		
		logFC	raw *p*	adj. *p*	logFC	raw *p*	adj. *p*
FBgn0050029	CR30029	-1.56	2.0E-06	8.0E-04	-1.46	2.2E-13	5.3E-11
FBgn0002121	l(2)gl	-0.36	8.9E-06	1.6E-03	-1.22	1.3E-13	4.1E-11
FBgn0035811	CG12262	-0.50	1.8E-05	2.3E-03	-0.70	2.2E-12	2.0E-10
FBgn0030484	GstT4	-0.59	3.5E-05	3.3E-03	-1.11	3.1E-12	2.5E-10
FBgn0027581	CG6191	-1.36	4.5E-05	3.7E-03	-0.81	2.6E-12	2.2E-10
FBgn0027601	pdgy	-0.59	5.3E-05	3.9E-03	-1.42	1.7E-12	1.8E-10
FBgn0014184	Oda	-0.32	7.6E-05	5.0E-03	-0.65	1.9E-12	1.8E-10
FBgn0015222	Fer1HCH	-0.52	1.8E-04	8.0E-03	-0.85	3.4E-12	2.6E-10
FBgn0260462	CG12163	-0.31	2.0E-04	8.5E-03	-0.66	5.1E-12	3.5E-10
FBgn0027572	CG5009	-0.41	3.0E-04	1.0E-02	-1.23	6.1E-12	3.9E-10
FBgn0030519	CG11151	-0.34	3.7E-04	1.2E-02	-1.30	1.7E-11	7.6E-10
FBgn0032900	CG14401	-0.39	3.3E-04	1.1E-02	-1.23	2.0E-11	8.7E-10
FBgn0035094	CG9380	-0.59	3.9E-04	1.2E-02	-1.00	3.2E-11	1.2E-09
FBgn0036945	Ssk	-0.45	3.3E-04	1.1E-02	-0.89	3.7E-11	1.3E-09
FBgn0040398	CG14629	-0.44	3.5E-04	1.1E-02	-0.97	4.4E-11	1.5E-09
FBgn0086346	ALiX	-0.29	2.5E-04	9.8E-03	-0.78	6.0E-11	1.8E-09
FBgn0031213	galectin	-0.28	4.4E-04	1.3E-02	-0.65	2.0E-10	4.6E-09
FBgn0039209	REPTOR	-0.27	4.9E-04	1.3E-02	-0.56	2.8E-10	5.7E-09
FBgn0035499	Chd64	-0.21	5.4E-04	1.4E-02	-0.74	2.9E-10	5.9E-09
FBgn0036337	AdenoK	-0.30	6.6E-04	1.6E-02	-0.50	3.9E-10	7.0E-09
FBgn0050035	Tret1-1	-0.37	6.9E-04	1.6E-02	-0.84	6.6E-10	1.0E-08
FBgn0033945	CG12868	-0.53	8.3E-04	1.8E-02	-0.36	8.8E-10	1.2E-08
FBgn0005278	Sam-S	-0.37	9.2E-04	1.9E-02	-0.67	9.8E-10	1.3E-08
FBgn0029504	CHES-1-like	-0.36	1.9E-03	2.8E-02	-0.65	1.4E-09	1.6E-08
FBgn0032940	Mio	-0.23	2.0E-03	2.8E-02	-0.36	1.5E-09	1.7E-08

FB Gene ID	Symbol	Selected – Control			*Acetobacter* – GF		
		**logFC**	**raw *p***	**adj. *p***	**logFC**	**raw *p***	**adj. *p***
FBgn0032603	CG17928	-0.96	2.1E-03	2.9E-02	-0.92	1.5E-09	1.7E-08
FBgn0014930	CG2846	-0.32	2.2E-03	3.0E-02	-0.81	1.8E-09	1.9E-08
FBgn0050011	gem	-0.17	2.4E-03	3.1E-02	-0.37	3.2E-09	2.8E-08
FBgn0036821	CG3961	-0.60	2.9E-03	3.5E-02	-0.53	3.1E-09	2.8E-08
FBgn0051352	Unc-115a	-0.26	3.0E-03	3.5E-02	-0.46	4.0E-09	3.3E-08
FBgn0033127	Tsp42Ef	-0.23	3.2E-03	3.7E-02	-0.41	7.5E-09	5.4E-08
FBgn0013576	mtd	-0.21	3.4E-03	3.8E-02	2.60	9.7E-09	6.5E-08
FBgn0040532	CG8369	-0.30	4.2E-03	4.2E-02	1.87	1.1E-08	7.1E-08
FBgn0013770	Cp1	-0.32	4.4E-03	4.4E-02	-0.42	1.5E-08	9.1E-08
FBgn0026415	Idgf4	-0.27	4.7E-03	4.5E-02	-0.30	4.2E-08	2.0E-07
FBgn0039266	CG11791	-0.19	4.7E-03	4.5E-02	-0.49	5.0E-08	2.4E-07
FBgn0026787	Nhe1	-0.19	4.9E-03	4.7E-02	-0.72	6.0E-08	2.7E-07
FBgn0001612	Grip91	0.16	5.2E-03	4.8E-02	-0.79	6.8E-08	3.1E-07
FBgn0050022	CG30022	-0.44	5.7E-03	5.0E-02	-0.57	8.9E-08	3.9E-07
FBgn0263773	fok	-0.36	6.0E-03	5.1E-02	-0.34	1.3E-07	5.2E-07
FBgn0038194	Cyp6d5	-0.63	7.2E-03	5.6E-02	-0.45	1.5E-07	6.0E-07
FBgn0020503	CLIP-190	-0.19	7.6E-03	5.8E-02	-0.51	1.8E-07	7.1E-07
FBgn0033465	Etf-QO	-0.38	7.7E-03	5.8E-02	-0.54	3.3E-07	1.2E-06
FBgn0017581	Lk6	-0.24	8.5E-03	6.2E-02	-0.58	3.3E-07	1.2E-06
FBgn0034035	CG8207	-0.20	9.7E-03	6.6E-02	0.76	4.2E-07	1.5E-06
FBgn0033521	CG12896	3.30	1.1E-02	7.1E-02	0.26	5.0E-07	1.7E-06
FBgn0050055	CR30055	0.24	1.1E-02	7.1E-02	-0.92	5.3E-07	1.8E-06
FBgn0067783	att-ORFA	-0.34	1.1E-02	7.1E-02	0.60	6.0E-07	2.0E-06
FBgn0265935	coro	-0.18	1.2E-02	7.4E-02	-0.33	7.6E-07	2.5E-06
FBgn0016917	Stat92E	-0.16	1.4E-02	8.1E-02	-0.61	8.9E-07	2.9E-06
FBgn0261618	larp	-0.17	1.7E-02	8.8E-02	-1.18	1.1E-06	3.6E-06
FBgn0051858	t-cup	0.97	1.9E-02	9.4E-02	-0.36	1.2E-06	3.8E-06
FBgn0027280	l(1)G0193	-0.18	2.0E-02	9.6E-02	-0.36	1.4E-06	4.4E-06
FBgn0032243	Klp31E	-0.16	1.9E-02	9.5E-02	2.44	2.8E-06	8.1E-06

FB Gene ID	Symbol	Selected – Control			*Acetobacter* – GF		
		logFC	raw *p*	adj. *p*	logFC	raw *p*	adj. *p*
FBgn0041342	Cct1	-0.16	2.0E-02	9.6E-02	-1.25	3.2E-06	9.1E-06
FBgn0024945	NitFhit	-0.17	2.2E-02	1.0E-01	0.24	4.2E-06	1.2E-05
FBgn0020249	stck	-0.15	2.3E-02	1.0E-01	-0.79	4.5E-06	1.2E-05
FBgn0016031	lama	-0.30	2.6E-02	1.1E-01	-0.30	4.8E-06	1.3E-05
FBgn0004401	Pep	0.11	2.9E-02	1.2E-01	-0.23	7.3E-06	1.9E-05
FBgn0020309	crol	0.15	3.3E-02	1.2E-01	0.38	7.8E-06	2.1E-05
FBgn0037834	Art1	0.15	3.3E-02	1.2E-01	-0.26	9.7E-06	2.5E-05
FBgn0029820	CG16721	-0.21	4.2E-02	1.4E-01	-0.33	1.1E-05	2.8E-05
FBgn0004395	unk	0.35	4.8E-02	1.5E-01	-0.87	1.1E-05	2.8E-05
FBgn0262736	Vha16-1	-0.21	5.0E-02	1.5E-01	-0.29	1.3E-05	3.2E-05
FBgn0014037	Su(Tpl)	-0.08	5.2E-02	1.6E-01	-0.47	1.5E-05	3.8E-05
FBgn0034736	CG6018	-0.37	5.4E-02	1.6E-01	-0.23	2.6E-05	6.1E-05
FBgn0037955	CG6950	-0.26	5.6E-02	1.6E-01	0.54	3.7E-05	8.6E-05
FBgn0025592	Gyk	-0.26	5.8E-02	1.7E-01	-0.29	4.4E-05	1.0E-04
FBgn0001186	Hex-A	-0.21	7.3E-02	1.9E-01	0.24	4.5E-05	1.0E-04
FBgn0030966	CG7280	-0.19	7.6E-02	1.9E-01	-0.25	4.7E-05	1.1E-04
FBgn0050403	CG30403	0.23	7.7E-02	1.9E-01	-0.23	6.3E-05	1.4E-04
FBgn0031228	CG11455	-0.16	8.6E-02	2.1E-01	0.23	7.1E-05	1.6E-04
FBgn0022029	l(2)k01209	-0.08	1.1E-01	2.4E-01	-0.23	7.4E-05	1.6E-04
FBgn0013733	shot	-0.18	1.2E-01	2.6E-01	-0.36	8.0E-05	1.7E-04
FBgn0037011	CG4858	-0.12	1.2E-01	2.6E-01	-0.20	1.1E-04	2.3E-04
FBgn0262475	bru-2	-0.12	1.2E-01	2.6E-01	-0.30	1.8E-04	3.6E-04
FBgn0034479	CG8654	-0.38	1.3E-01	2.7E-01	-0.17	2.0E-04	4.1E-04
FBgn0031914	CG5973	-0.22	1.4E-01	2.8E-01	-0.22	2.4E-04	4.8E-04
FBgn0037239	CG11739	-0.17	1.5E-01	2.9E-01	0.30	2.6E-04	5.1E-04
FBgn0035142	hipk	0.13	1.8E-01	3.2E-01	0.16	2.9E-04	5.7E-04
FBgn0004397	Vinc	-0.11	1.8E-01	3.3E-01	-0.15	3.2E-04	6.2E-04
FBgn0038922	CG6439	-0.15	1.9E-01	3.3E-01	-0.17	3.2E-04	6.3E-04
FBgn0033247	Nup44A	0.08	1.9E-01	3.4E-01	0.54	3.3E-04	6.3E-04

FB Gene ID	Symbol	Selected – Control			*Acetobacter* – GF		
		logFC	raw *p*	adj. *p*	logFC	raw *p*	adj. *p*
FBgn0034214	CG6550	-0.09	1.9E-01	3.4E-01	-0.45	4.7E-04	8.8E-04
FBgn0039857	RpL6	-0.07	1.9E-01	3.4E-01	-0.19	5.4E-04	1.0E-03
FBgn0032699	CG10383	-0.13	2.3E-01	3.9E-01	-0.22	8.2E-04	1.5E-03
FBgn0015834	Trip1	-0.06	2.5E-01	4.0E-01	-0.63	8.4E-04	1.5E-03
FBgn0034691	Synj	0.06	2.5E-01	4.1E-01	0.15	1.1E-03	1.9E-03
FBgn0033188	Drat	-0.10	2.7E-01	4.3E-01	0.20	1.5E-03	2.6E-03
FBgn0041210	HDAC4	-0.09	2.7E-01	4.3E-01	0.19	1.5E-03	2.6E-03
FBgn0051324	CG31324	-0.18	2.7E-01	4.3E-01	-0.24	1.9E-03	3.2E-03
FBgn0261560	Thor	-0.18	2.7E-01	4.2E-01	-0.28	2.1E-03	3.5E-03
FBgn0030520	Pdcd4	0.17	2.8E-01	4.4E-01	-0.16	2.1E-03	3.6E-03
FBgn0029785	RpL35	-0.05	3.1E-01	4.7E-01	-0.15	2.6E-03	4.4E-03
FBgn0032945	CG8665	-0.28	3.2E-01	4.8E-01	-0.36	4.0E-03	6.4E-03
FBgn0037195	CG11226	0.29	3.2E-01	4.8E-01	0.28	4.0E-03	6.5E-03
FBgn0085485	CG34456	-0.23	3.1E-01	4.7E-01	-2.74	5.2E-03	8.2E-03
FBgn0005670	Cyp4d1	-0.12	3.8E-01	5.4E-01	0.23	5.6E-03	8.9E-03
FBgn0030733	CG3560	-0.10	4.0E-01	5.6E-01	0.16	7.8E-03	1.2E-02
FBgn0052865	CR32865	-0.19	4.1E-01	5.7E-01	-0.13	1.1E-02	1.6E-02
FBgn0262656	dm	-0.07	4.1E-01	5.7E-01	-0.25	1.2E-02	1.8E-02
FBgn0023522	CG11596	-0.04	4.2E-01	5.8E-01	-0.31	1.7E-02	2.5E-02
FBgn0029915	CG14434	0.05	4.3E-01	5.8E-01	0.09	2.0E-02	2.8E-02
FBgn0034748	CG17807	-0.06	4.4E-01	6.0E-01	-0.17	2.1E-02	2.9E-02
FBgn0037561	CG9630	-0.06	4.5E-01	6.1E-01	0.16	2.3E-02	3.3E-02
FBgn0261239	Hr39	0.07	4.7E-01	6.2E-01	-0.40	5.4E-02	7.1E-02
FBgn0036366	CG10133	-0.06	4.8E-01	6.3E-01	0.14	5.7E-02	7.5E-02
FBgn0033919	CG8547	-0.04	5.2E-01	6.6E-01	-0.15	5.8E-02	7.6E-02
FBgn0016977	spen	0.04	5.5E-01	6.9E-01	-0.11	6.9E-02	8.9E-02
FBgn0036030	CG6767	-0.04	5.5E-01	6.9E-01	-0.13	7.8E-02	1.0E-01
FBgn0015602	BEAF-32	0.04	5.7E-01	7.1E-01	-0.08	7.9E-02	1.0E-01
FBgn0025574	Pli	-0.08	5.7E-01	7.1E-01	-0.17	8.3E-02	1.1E-01

FB Gene ID	Symbol	Selected – Control			*Acetobacter* – GF		
		logFC	raw *p*	adj. *p*	logFC	raw *p*	adj. *p*
FBgn0037235	CG1103	0.07	5.7E-01	7.0E-01	-0.09	9.0E-02	1.1E-01
FBgn0053138	AGBE	0.08	5.7E-01	7.1E-01	-0.08	1.1E-01	1.4E-01
FBgn0016930	Dyrk2	0.10	5.8E-01	7.2E-01	-0.09	1.2E-01	1.5E-01
FBgn0033725	Cpr49Ac	0.27	6.2E-01	7.4E-01	-0.13	1.4E-01	1.8E-01
FBgn0030261	CG15203	-0.06	6.5E-01	7.7E-01	-0.17	1.4E-01	1.7E-01
FBgn0037635	CG9837	-0.05	6.8E-01	7.9E-01	-0.07	1.5E-01	1.9E-01
FBgn0004396	CrebA	-0.03	7.4E-01	8.4E-01	-0.23	2.0E-01	2.4E-01
FBgn0020386	Pdk1	-0.02	7.4E-01	8.3E-01	-0.08	2.2E-01	2.6E-01
FBgn0261444	CG3638	0.02	7.7E-01	8.6E-01	0.05	2.5E-01	2.9E-01
FBgn0016076	vri	0.04	7.8E-01	8.6E-01	-0.03	2.7E-01	3.2E-01
FBgn0037526	CG10092	0.02	8.2E-01	8.9E-01	-0.07	2.9E-01	3.3E-01
FBgn0052576	CG32576	-0.01	8.4E-01	9.0E-01	-0.05	3.5E-01	4.0E-01
FBgn0037891	CG5214	-0.02	8.8E-01	9.3E-01	-0.09	3.9E-01	4.3E-01
FBgn0036134	fd68A	0.01	8.9E-01	9.3E-01	0.12	4.9E-01	5.3E-01
FBgn0265623	Su(z)2	-0.01	9.0E-01	9.4E-01	0.05	6.3E-01	6.7E-01
FBgn0033240	CG2906	-0.01	9.1E-01	9.5E-01	-0.02	6.8E-01	7.2E-01
FBgn0086758	chinmo	0.01	9.2E-01	9.5E-01	0.08	6.9E-01	7.3E-01
FBgn0000250	cact	0.00	9.3E-01	9.6E-01	0.01	7.7E-01	8.0E-01
FBgn0037150	CG7133	0.00	9.6E-01	9.8E-01	-0.01	7.9E-01	8.2E-01
FBgn0050349	CG30349	0.00	1.0E+00	1.0E+00	0.02	8.6E-01	8.8E-01
